# Evaluation of blue and red two-tone colored staining solution for dental plaque imaging using an intraoral scanner

**DOI:** 10.4317/jced.61808

**Published:** 2024-07-01

**Authors:** Chihiro Yoshiga, Reiko Kobatake, Kazuya Doi, Hiroshi Oue, Maiko Kawagoe, Kazuhiro Tsuga

**Affiliations:** 1 Department of Advanced Prosthodontics, Hiroshima University Graduate School of Biomedical and Health Sciences, Hiroshima, Japan; 2 Department of Dental hygiene, Hiroshima University Hospital, Hiroshima, Japan

## Abstract

**Background:**

We first reported that intraoral imaging with a color-imaging intraoral scanner could evaluate stained plaques and contribute to oral hygiene instructions. In this study, we aimed to evaluate the effects of red and blue staining on dental hygiene assessment with an intraoral scanner using plaque control record values and plaque-stained areas.

**Material and Methods:**

Fifteen patients (six males and nine females, aged 25–85 years) were included in this study. The patients’ teeth were stained with a two-tone (blue and red) dental plaque staining solution, and images of their teeth were recorded with an intraoral scanner and a digital camera. The plaque control record was measured by direct viewing, as usual, and on a monitor using intraoral scanner image. In addition, the plaque deposition area was measured using images obtained using an intraoral scanner and images taken by a digital camera.

**Results:**

Most parts were stained red and blue dental plaque staining was also observed. Plaque control record values tended to be higher in the intraoral scanner evaluation than in the direct evaluation. The plaque-stained area was larger in intraoral scanner images than in camera images.

**Conclusions:**

It is possible to use a two-tone plaque-staining solution for oral health evaluation using an intraoral scanner. In the future, we need to investigate cases of blue-stained plaques.

** Key words:**Intraoral scanner, oral hygiene, dental plaque, preventive dentistry.

## Introduction

Intra oral scanners (IOS) are increasingly utilized in the fabrication of prosthetics such as computer-aided design and computer-aided manufacturing of crowns, garnering significant attention in recent years (1-4). We first reported that intraoral imaging with a color imaging IOS can evaluate stained plaques and contribute to oral hygiene instructions (5). Dental plaque is a major risk factor for dental caries and periodontal diseases (6). Therefore, it is necessary to maintain effective plaque control through oral hygiene instruction, which involves clearly visualizing dental plaque using a plaque disclosing solution (7).

Utilizing an IOS enables visualization of areas that are difficult to see directly or with just a mirror. The images can be enlarged and rotated on the screen, facilitating the surgeon’s explanation to the patient and enhancing the patient’s comprehension of their oral hygiene status (8-10). A comparison between IOS and direct intraoral assessment using O’Reilly’s Plaque Control Record (PCR) revealed higher PCR values with IOS in assessing healthy teeth (11). Subsequent findings indicated that IOS yielded higher PCR values compared to direct viewing method in clinical cases. However, on examination of the stained area of dental plaque in IOS and camera images, the stained area appeared slightly larger in the IOS images (12). Conversely, no difference was observed in PCR measurements of anterior teeth between IOS and direct viewing (11), possibly due to direct visibility of these areas, facilitating accurate PCR measurements. Therefore, despite slightly higher levels of stained dental plaque compared to direct observation and cameras, it is clearly confirmed in IOS, suggesting the utility of oral hygiene evaluation with IOS.

The pigments in the stains used in our clinical practice bind to the proteins and polysaccharides in the plaque, resulting in colored stains (13,14). Commonly used colors include red and blue. Red stains such as phloxine and rose bengal are generally used to stain early-stage plaques owing to the large dye particles (15). In contrast, the brilliant blue dye is utilized in blue stain, targeting only old plaque. Its small particles penetrate the tight spaces within accumulated old plaque, avoiding less dense areas, such as new plaque (13).

Previous studies used red-colored dyes, which pose challenges in distinguishing them from gingival color in IOS due to low pixel counts compared with cameras, particularly in the tooth marginal area (12). Moreover, no clinical studies have examined the effect of plaque staining color on oral hygiene evaluation using IOS.

This study aimed to evaluate the effect of red and blue staining on dental hygiene assessment with IOS, using PCR values and plaque-stained areas.

## Material and Methods

-Study design and participants

This was an interventional, single-arm, open-label, uncontrolled, single-comparison study, approved by the Hiroshima University Hospital Accreditation Review Board (CRB 6180006) and conducted as a specific clinical study (jRCTs 062220068) at Hiroshima University Hospital School of Dentistry from January to November 2023, according to the Good Clinical Practice guidelines of the International Conference on Harmonization.

Fifteen patients (six males and nine females, aged 25–85 years) undergoing regular oral hygiene were enrolled. Each participant provided informed consent by signature. Inclusion criteria were as follows: 1) age 18 years or older; 2) written consent for participation; 3) periodontal disease and/or peri-implantitis; 4) regular oral hygiene for at least 6 months by a dentist or dental hygienist; 5) PCR of more than 20%; and 6) cognitive ability to understand and answer the questionnaire questions accurately.

Exclusion criteria were as follow: 1) patients with a pacemaker or implanTable cardioverter-defibrillator (ICD); 2) edentulous jaw; 3) missing all molars and bicuspids; 4) patients with oral dysphonia or TMJ disorder; and 5) patients deemed inappropriate by the principal investigator or principal study investigator.

-Study procedures

The participants visited the hospital under normal oral hygiene conditions and were briefed on the study procedures. Their teeth were then stained with a two-tone (blue and red) dental plaque staining solution (PROSPEC; GC Corporation, Tokyo, Japan). After staining with a cotton ball soaked in the solution, participants rinsed their mouths with water. Subsequently, the oral cavity was dried well, and both the intraoral cavity and buccal aspect of the molars were photographed using an IOS (TRIOS 3® Basic, 3Shape) and a digital SLR camera (EOS 60D; CANON), respectively.

-Evaluation of Dental Plaque Staining Condition

After completing intraoral imaging using the IOS and digital SLR camera, the PCR values were measured intraorally using a dental mirror for direct viewing, as usual, considered the direct evaluation. PCR values were also measured on a monitor using the photographed IOS images, utilized for IOS evaluation. PCR values were measured by dividing the crown into six areas: the proximal, central, and distal surfaces on the labial, buccal, and lingual or palatal sides. This assessment was conducted based on the presence of plaque deposits, and all measurements were performed by a dental hygienist with more than 15 years of experience.

-Comparison of PCR values

The PCR values obtained from direct and IOS evaluations were compared for the entire dentition, maxilla, maxillary anterior labial and palatal regions, maxillary buccal and palatal molars, mandible, mandibular anterior labial and lingual molars, and mandibular buccal and lingual molars. The Wilcoxon signed-rank test was used to evaluate PCR values in direct and IOS evaluations. Statistical analyses were performed using Prism v7 (GraphPad, La Jolla, CA), with the significance level set at *p* <0.05.

-Comparison of Dental Plaque Coverage

**Figure 1:**
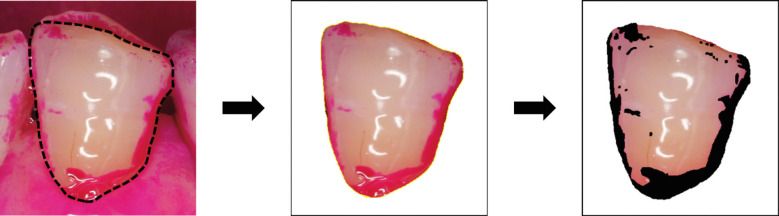
Evaluation of the plaque stained area. After trimming only the buccal side of the tooth as the region of interest, the stained area is marked and measured.

The plaque deposition area was measured using images obtained by the IOS and digital SLR camera. ImageJ software was used for area measurement, with the stained area measured after trimming the buccal side of the tooth (Fig. 1). The percentage of plaque deposition was calculated by dividing the plaque deposition area by the total tooth area. The digital SLR camera images were directly evaluated, while IOS images were evaluated using IOS evaluation methods. The Wilcoxon signed-rank test was used to compare plaque deposition area percentages. Statistical analyses were performed using Prism v7 (GraphPad, La Jolla, CA), with a significance level set at *p* <0.05.

## Results

Observation of plaque stained area

**Figure 2:**
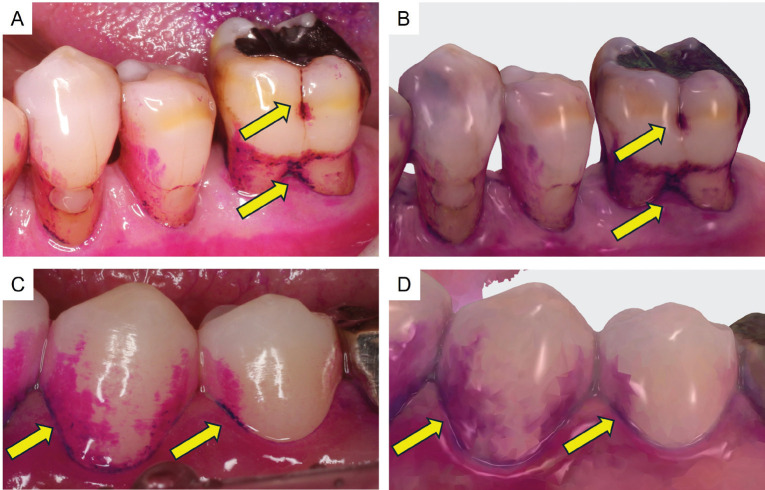
Plaque observations. A,C: camera image. B,D: IOS image. Most of the dental plaque stained red. Very few parts were stained blue (yellow arrow).

Some parts were stained blue (yellow arrow), while the majority stained red. Both the blue and red dental plaque stains were visible in the IOS images, with the blue stain appearing darker than the red stain (Fig. 2).

-Comparison of PCR values

PCR values obtained from IOS evaluation were higher than those from direct evaluation ( Table 1). Significant differences in PCR values were observed between the buccal and palatal molars in the maxilla, as well as between buccal and lingual sides of the mandible. However, no significant differences were noted in anterior buccal, palatal, or lingual values between the maxilla and mandible.

-Comparison of stained surface ratio

**Figure 3:**
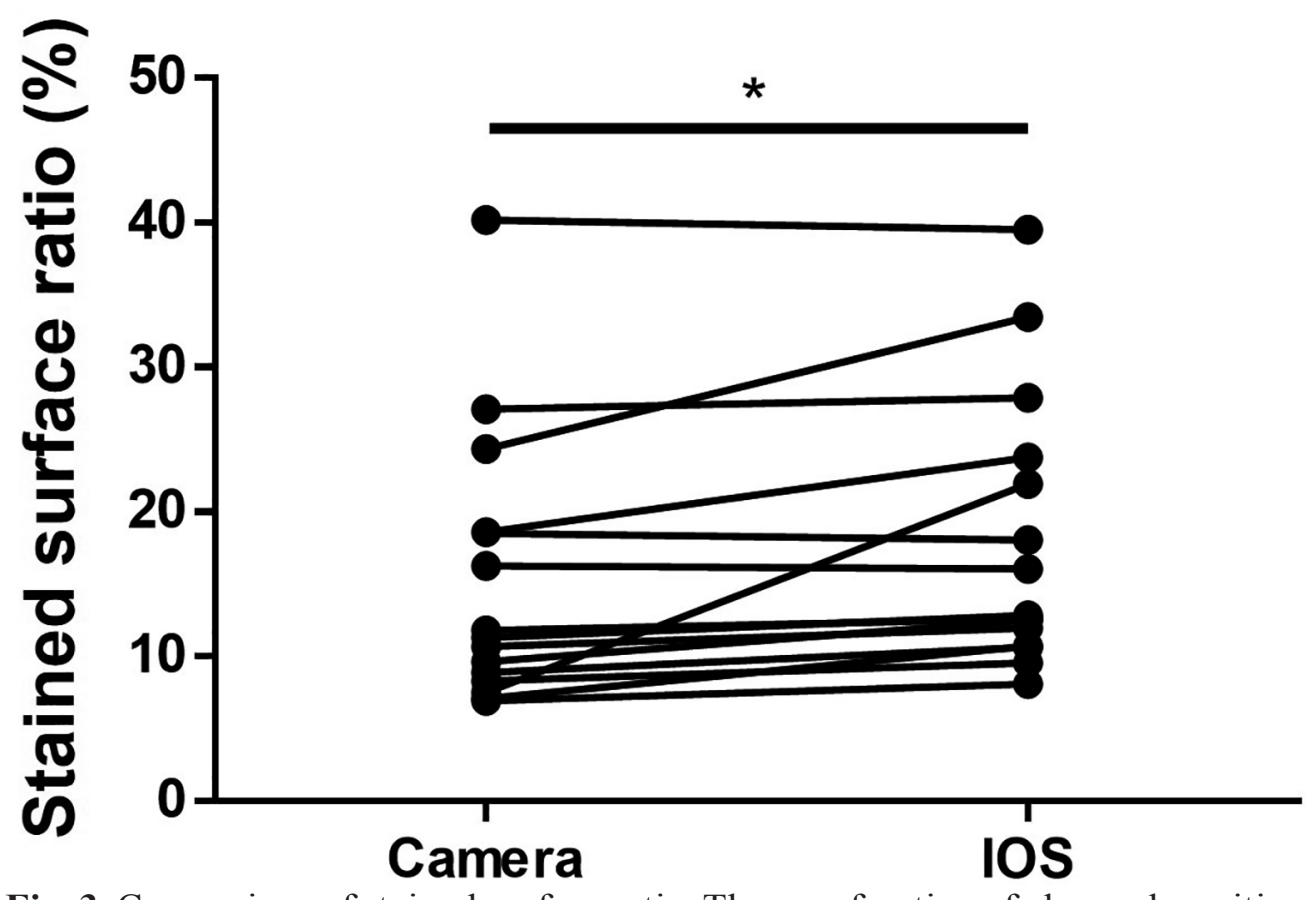
Comparison of stained surface ratio. The area fraction of plaque deposition is significantly higher in the IOS evaluation than in the direct evaluation.

In IOS images, the contours of plaque-stained areas appeared blurred, and the stained areas appeared wider than those in the camera images. The area fraction of plaque deposition was significantly higher in IOS evaluation than in direct evaluation (Fig. 3).

## Discussion

The usefulness of oral health evaluation through IOS has been reported in several studies (8,9) as it enables easy visualization of areas that are difficult to observe directly, effectively identifying plaque attachment sites.

In our previous studies, dental plaques were stained with a commonly used red plaque staining solution, followed by imaging with IOS, to assess the PCR value (5,11).

When for comparing plaque attachment sites stained with red staining solution using IOS and visual inspection, no significant differences were noted in the anterior teeth. However, for molars, PCR score by IOS was significantly higher on both the buccal and labial sides compared to direct observation (11). This was because it was difficult to observe details of the molars without a mirror and the image quality (number of pixels) was lower in the IOS than in the camera images.

In this study, we used a blue and red two-tone colored staining solution, commonly employed in clinical practice alongside the red colored solution. The two-tone approach facilitated the differentiation of new plaques (stained red) from old plaques (stained blue).

These three color attributes determine the color. “Hue,’’ which represents the difference in color tone such as red and blue, “lightness,’’ which represents the brightness of the color, and “saturation,’’ which represents the vividness of the color.

In the IOS images, the red-stained plaques were red, with high brightness and saturation. Conversely, the blue-stained plaques were blue, with low brightness and saturation. In the IOS images, the gingival color was brighter and more saturated than that of the red-stained plaque; however, the hues were notably similar.

Statistical evaluation of the difference in visual appearance between direct view and IOS revealed that there was no difference in the anterior view, and IOS was significantly higher in the molars. The two-tone color solution examination showed the same results as those in red, as described in our previous study (11).

There was a tendency for the two-tone color to have a higher overall *p-value*. A high *p-value* indicated that there was little significant difference between direct observation and IOS, and it was possible to confirm the plaque staining site with IOS.

In the case of red staining, the stained plaque had a color similar to that of the gums; therefore, it was difficult to distinguish the plaques on the marginal area.

In contrast, for the palatal side of the maxillary anterior teeth, buccal side of the maxillary molars, and labial/lingual side of the mandibular anterior teeth, the *p-value*s tended to be lower for the two-tone than for red. Owing to the limited number of blue-stained areas observed in this study, it was challenging to thoroughly discuss the results.

One advantage of IOS is its ability to evaluate both PCR and the area ratio, as demonstrated in several studies (16,17). Assessing the area provides an absolute evaluation and allows for a more accurate depiction of oral hygiene status. This evaluation was performed by displaying one camera image and one IOS image captured from the same angle on a monitor as images of the same size. Therefore, there is no effect on enlargement and rotation processing, which is one of the advantages of IOS.

The plaque adhesion rate measured via IOS evaluation was higher than that observed in camera imaging, consistent with previous findings using a red plaque staining solution.

We attribute this to image the quality of the IOS, which, in this study, had lower resolution (1280 × 870 pixels) compared to the camera (1980 × 1080 pixels). Consequently, the larger pixel size in IOS images led to a spread-out appearance of stained areas, regardless of the color. Conversely, the high image quality and small pixel size of the camera produced relatively clear images, enabling more detailed evaluation.

Furthermore, the distinct hue of the blue staining facilitated differentiation between cervical plaque and the gingival boundary.

However, due to predominance of red-stained areas and limited presence of old plaques in this study, there were challenges in evaluating the blue-stained areas. Participants received regular oral hygiene instructions, resulting in fewer old plaque adhesions.

These results indicate the potential utility of a two-tone plaque staining solution for oral health evaluation using IOS. Future research should focus on cases involving blue-stained plaques to further elucidate its effectiveness.

## Figures and Tables

**Table 1:** 

Entire	Maxillary				Mandibular			
	Anterior teeth		Posterior teeth		Anterior teeth		Posterior teeth	
	Labial	Palatal	Buccal	Palatal	Labial	Lingual	Buccal	Lingual
IOS*>Direct P=0.0009	No significance P=0.53	No significance P=0.052	IOS*>Direct P=0.0002	IOS*>Direct P=0.0005	No significance P=0.064	No significance P=0.16	IOS*>Direct P=0.0034	IOS*>Direct P=0.042

## Data Availability

The datasets used and/or analyzed during the current study are available from the corresponding author.
